# Bioactive Compounds from a Gorgonian Coral *Echinomuricea* sp. (Plexauridae)

**DOI:** 10.3390/md10051169

**Published:** 2012-05-23

**Authors:** Hsu-Ming Chung, Pei-Han Hong, Jui-Hsin Su, Tsong-Long Hwang, Mei-Chin Lu, Lee-Shing Fang, Yang-Chang Wu, Jan-Jung Li, Jih-Jung Chen, Wei-Hsien Wang, Ping-Jyun Sung

**Affiliations:** 1 Department of Marine Biotechnology and Resources, National Sun Yat-sen University, Kaohsiung 804, Taiwan; Email: shiuanmin@yahoo.com.tw; 2 National Museum of Marine Biology and Aquarium, Pingtung 944, Taiwan; Email: peihan520@yahoo.com.tw (P.-H.H.); x2219@nmmba.gov.tw (J.-H.S.); jinx6609@nmmba.gov.tw (M.-C.L.); jj@nmmba.gov.tw (J.-J.L.); 3 Graduate Institute of Marine Biotechnology, National Dong Hwa University, Pingtung 944, Taiwan; 4 Division of Marine Biotechnology, Asia-Pacific Ocean Research Center, National Sun Yat-sen University, Kaohsiung 804, Taiwan; 5 Graduate Institute of Natural Products, Chang Gung University, Taoyuan 333, Taiwan; Email: htl@mail.cgu.edu.tw; 6 Department of Sport, Health and Leisure, Cheng Shiu University, Kaohsiung 833, Taiwan; Email: lsfang@csu.edu.tw; 7 School of Chinese Medicine, College of Chinese Medicine, China Medical University, Taichung 404, Taiwan; Email: yachwu@mail.cmu.edu.tw; 8 Natural Medicinal Products Research Center, China Medical University Hospital, Taichung 404, Taiwan; 9 Center for Molecular Medicine, China Medical University Hospital, Taichung 404, Taiwan; 10 Department of Pharmacy and Graduate Institute of Pharmaceutical Technology, Tajen University, Pingtung 907, Taiwan; Email: jjchen@mail.tajen.edu.tw; 11 Department of Life Science and Institute of Biotechnology, National Dong Hwa University, Hualien 974, Taiwan

**Keywords:** *Echinomuricea*, echinolabdane, yonarasterol, superoxide anion, elastase

## Abstract

A new labdane-type diterpenoid, echinolabdane A (**1**), and a new sterol, 6-*epi*-yonarasterol B (**2**), were isolated from a gorgonian coral identified as *Echinomuricea* sp. The structures of metabolites **1** and **2** were elucidated by spectroscopic methods. Echinolabdane A (**1**) possesses a novel tetracyclic skeleton with an oxepane ring jointed to an α,β-unsaturated-γ-lactone ring by a hemiketal moiety, and this compound is the first labdane-type diterpenoid to be obtained from marine organisms belonging to the phylum Cnidaria. 6-*epi*-Yonarasterol B (**2**) is the first steroid derivative to be isolated from gorgonian coral belonging to the genus *Echinomuricea*, and this compound displayed significant inhibitory effects on the generation of superoxide anions and the release of elastase by human neutrophils.

## 1. Introduction

**Figure 1:**
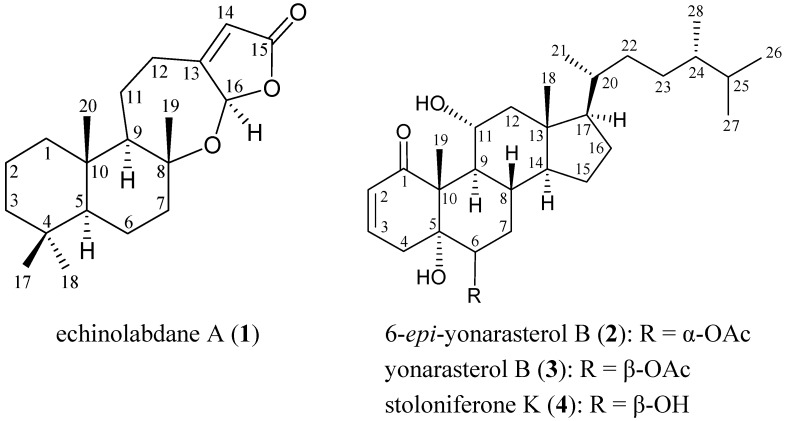
The structures of echinolabdane A (**1**), 6-*epi*-yonarasterol B (**2**), yonarasterol B (**3**) and stoloniferone K (**4**).

The search for new natural products from marine organisms has been remarkably successful, and gorgonian corals have been proven to be rich sources of interesting natural terpenoid derivatives [1,2]. In a previous study, two sesquiterpenoid phenols, (7*S*,10*R*)-(+)-10,11-epoxycurcuphenol and (+)-curcuphenol [3], were isolated from the Formosan gorgonian coral *Echinomuricea* sp. (family Plexauridae). In continuation of our search for new natural substances from marine invertebrates collected off the waters of Taiwan at the intersection point of the Kuroshio current and the South China Sea surface current, we have further isolated a new labdane-type diterpenoid, echinolabdane A (**1**), and a new steroid derivative, 6-*epi*-yonarasterol B (**2**), from *Echinomuricea* sp. In this paper, we describe the isolation, structural characterization and bioactivity of new compounds **1** and **2** (Figure 1).

## 2. Results and Discussion

Echinolabdane A (**1**) was isolated as an oil, and the molecular formula for this compound was determined using HRESIMS to be C_20_H_30_O_3_ (6° of unsaturation) (*m/z* 341.2095 [M + Na]^+^, calculated as 341.2093). An IR absorption at 1765 cm^−1^ suggested the presence of a γ-lactone group in **1**. The ^13^C NMR data for **1** confirmed the presence of 20 carbon signals (Table 1), which were characterized by DEPT as four methyls, seven sp^3^ methylenes, three sp^3^ methines, an sp^2^ methine, three sp^3^ quaternary carbons and two sp^2^ quaternary carbons. A suite of resonances at *δ*_C_ 170.6 (C-15), 169.3 (C-13), 117.0 (CH-14) and 100.9 (CH-16) could be assigned to the α,β-unsaturated-γ-lactone moiety. From the reported data, the carbon skeleton of **1** was suggested to be a diterpenoid derivative with four rings.

**Table 1:** ^1^H (400 MHz, CDCl_3_) and ^13^C (100 MHz, CDCl_3_) NMR data, ^1^H–^1^H COSY and HMBC correlations for diterpenoid **1**.

Position	*δ*_Η_ (*J* in Hz)	*δ*_C_, Mult.	^1^H–^1^H COSY	HMBC (H→C)
1a	1.69 m	40.6, CH_2_	H-1b, H_2_-2	C-10
1b	1.91 m		H-1a, H_2_-2	C-9
2a	1.45 m	18.6, CH_2_	H_2_-1, H-2b, H_2_-3	n.o.
2b	1.63 m		H_2_-1, H-2a, H_2_-3	n.o.
3a	1.16 dd(13.6, 4.0)	41.7, CH_2_	H_2_-2, H-3b	C-4, -17
3b	1.39 m		H_2_-2, H-3a	n.o.
4		33.4, C		
5	0.92 dd (9.6, 2.0)	56.1, CH	H_2_-6	C-4, -6, -10
6a	1.29 m	19.9, CH_2_	H-5, H-6b, H_2_-7	C-8, -10
6b	1.72 m		H-5, H-6a, H_2_-7	C-5, -8, -10
7a	0.94 m	39.8, CH_2_	H_2_-6, H-7b	C-6
7b	1.78 br d (11.6)		H_2_-6, H-7a	C-8
8		82.5, C		
9	1.38 m	60.6, CH	H_2_-11	C-8, -10, -11, -20
10		39.2, C		
11a	1.51 m	22.1, CH_2_	H-9, H-11b, H_2_-12	C-9
11b	1.94 m		H-9, H-11a, H_2_-12	C-8, -9
12a	2.22 m	29.3, CH_2_	H_2_-11, H-12b, H-14	C-13, -14
12b	2.91 ddd (13.6, 3.2, 2.4)		H_2_-11, H-12a	n.o.
13		169.3, C		
14	5.82 br s	117.0, CH	H-12a	C-12, -13, -15, -16
15		170.6, C		
16	6.07 s	100.9, CH		C-8, -13, -14, -15
17	0.89 s	33.4, CH_3_		C-3, -4, -5, -18
18	0.80 s	21.4, CH_3_		C-3, -4, -5, -17
19	1.25 s	22.3, CH_3_		C-7, -8, -9
20	0.78 s	15.6, CH_3_		C-1, -5, -9, -10

n.o. = not observed.

**Figure 2:**
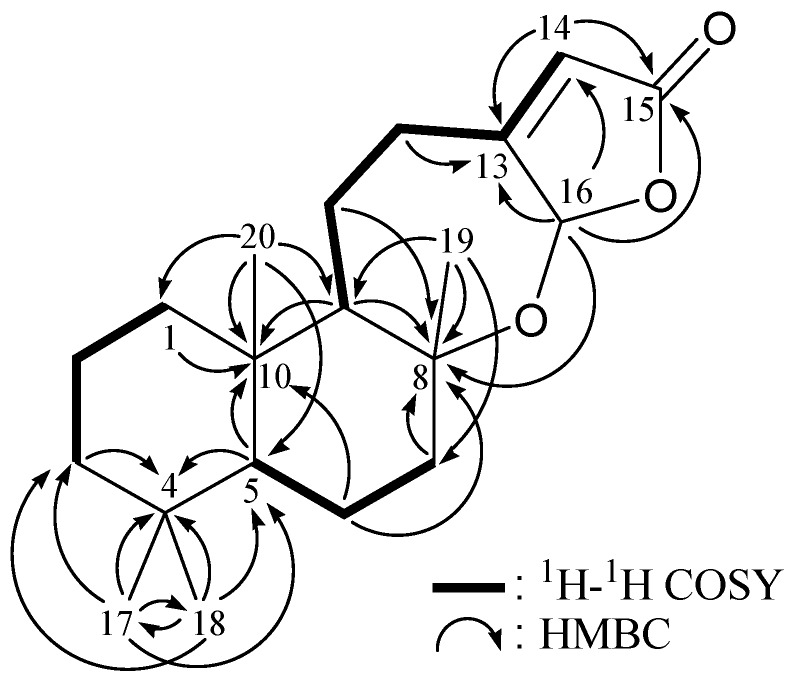
The ^1^H–^1^H COSY and selective key HMBC correlations for **1**.

From the ^1^H–^1^H COSY analysis of **1** (Table 1 and Figure 2), it was possible to establish the spin systems that map out the proton sequences from H_2_-1/H_2_-2/H_2_-3, H-5/H_2_-6/H_2_-7, H-9/H_2_-11/H_2_-12 and H-12a/H-14 (by allylic coupling), which was accomplished with the assistance of an HMBC experiment (Table 1 and Figure 2). The key HMBC correlations between the protons and quaternary carbons of **1**, including H-3a, H-5, H_3_-17, H_3_-18/C-4; H_2_-6, H-7b, H-9, H-11b, H-16, H_3_-19/C-8; H-1, H-5, H_2_-6, H-9, H_3_-20/C-10; H-12a, H-14, H-16/C-13; and H-14, H-16/C-15, permitted the elucidation of the carbon skeleton of **1**. The tertiary methyls at C-4, C-8 and C-10 were confirmed by the HMBC correlations between H_3_-17/C-3, C-4, C-5, C-18; H_3_-18/C-3, C-4, C-5, C-17; H_3_-19/C-7, C-8, C-9; and H_3_-20/C-1, C-5, C-9, C-10. Furthermore, an HMBC correlation between H-16 (*δ*_H_ 6.07) and an oxygenated quaternary carbon at *δ*_C_ 82.5 (C-8) suggested the presence of a C-8/16 ether linkage in **1**. The methine unit at *δ*_C_ 100.9 (CH-16) was more shielded than expected for an oxygenated C-atom and was correlated with the methine proton at *δ*_H_ 6.07 (H-16) in the HMQC spectrum, and this proton showed a ^2^*J*-correlation with C-13 and showed ^3^*J*-correlations with C-8, C-14 and C-15 in the HMBC spectrum, and was concluded to be a part of a hemiketal moiety.

**Figure 3:**
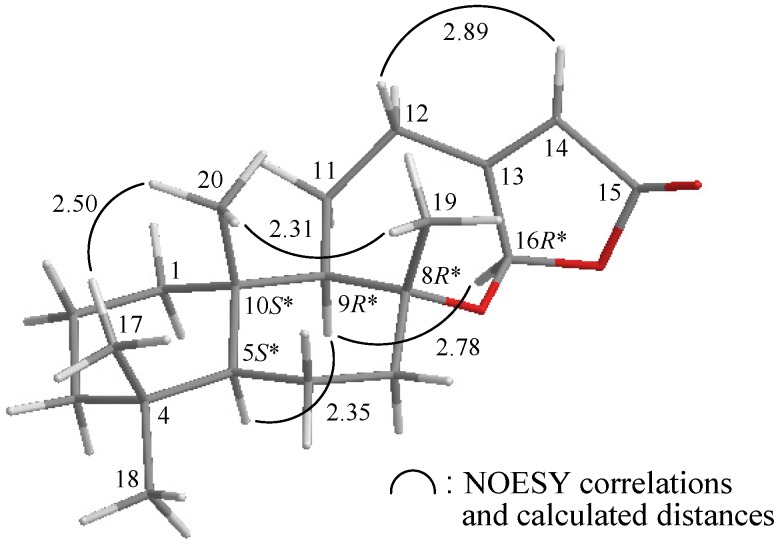
The computer-generated model of **1** using MM2 force field calculations and the calculated distances (**Å**) between selected protons with key NOESY correlations.

The relative configuration of **1** was elucidated mainly from a NOESY spectrum as being compatible with that of **1** offered by computer modeling (Figure 3) [4], in which the close contacts of atoms in space calculated were consistent with the NOESY correlations. In the NOESY analysis of **1**, the correlations of H-9 with H-5 and H-16, but not with H_3_-19 and H_3_-20, indicated that these protons (H-5, H-9 and H-16) were situated on the same face, and these were assigned as α protons, since the C-19 and C-20 methyls are β-substituents at C-8 and C-10, respectively. The *Z*-configuration of the C-13/14 double bond was elucidated from a correlation between H-14 (*δ*_H_ 5.82) and H-12b (*δ*_H_ 2.91). From the above evidence, the relative configurations of the chiral carbons of **1** were assumed to be 5S*, 8R*, 9R*, 10S* and 16R*. On the basis of the above findings, the structure of **1** was elucidated.

The *in vitro* cytotoxicity of labdane **1** was studied, and this compound exhibited weak cytotoxicity toward HL-60 (human acute promyelocytic leukemia) cells (IC_50_ = 19.1 μg/mL).

**Figure 4:**
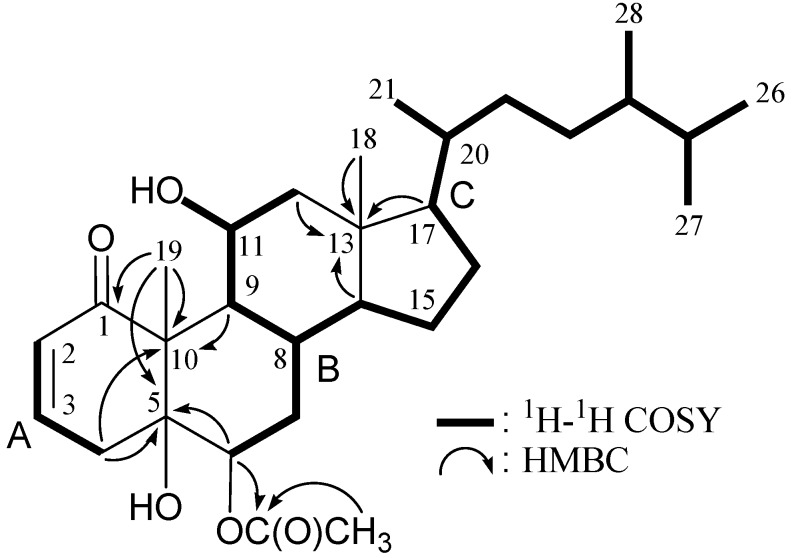
The ^1^H–^1^H COSY and selective key HMBC (protons→quaternary carbons) correlations for **2**.

6-*epi*-Yonarasterol B (**2**) had a molecular formula of C_30_H_48_O_5_ as indicated by HRESIMS at *m/z* 511.3396 (calculated for C_30_H_48_O_5_Na, 511.3399). The ^13^C NMR and DEPT spectra of **2** exhibited the presence of seven methyls, seven sp^3^ methylenes, nine sp^3^ methines, two sp^2^ methines, three sp^3^ quaternary carbons and two sp^2^ quaternary carbons (Table 2). The IR spectrum of **2** showed absorptions due to α,β-unsaturated ketone (1671 cm^−1^) and ester (1732 cm^−1^) groups. The presence of a conjugated enone system in **2** was also indicated by ^1^H (*δ*_H_ 6.68, 1H, ddd, *J* = 10.5, 5.5, 2.5 Hz, H-3; 6.15, 1H, dd, *J* = 10.5, 2.0 Hz, H-2) and ^13^C (*δ*_C_ 205.8, C-1; 140.7, CH-3; 128.8, CH-2) NMR spectra (Table 2). The presence of a secondary acetoxy group was evident from the IR (1732 cm^−1^), ^1^H (*δ*_H_ 2.11, 3H, s, acetate methyl; 5.06, 1H, dd, *J* = 12.0, 5.5 Hz, H-6) and ^13^C (*δ*_C_ 21.2, acetate methyl; 171.5, acetate carbonyl; 75.1, CH-6) NMR spectra. IR absorption at 3392 cm^−1^ and ^1^H NMR signals at *δ*_H_ 3.91 (1H, br s, H-11) and ^13^C NMR at *δ*_C_ 66.9 (CH-11) indicated the presence of a secondary hydroxy group. It was found that the structure of **2** is similar to the structures of known sterols yonarasterol B (**3**) [5] and stoloniferone K (**4**) [6] (Figure 1). All C-H correlations of **2** were detected in the HMQC experiment. The ^1^H–^1^H COSY spectrum exhibited partial structures A, B and C (Table 2 and Figure 4). In the HMBC spectrum, the partial structure A could be connected to B through three quaternary carbons C-1, C-5, C-10 and Me-19 (Table 2 and Figure 4). Partial structure B could be connected to C through the remaining quaternary carbon C-13 and Me-18. Based on the above findings, the planar structure of **2** was concluded, as shown in Figure 4. 

**Figure 5:**
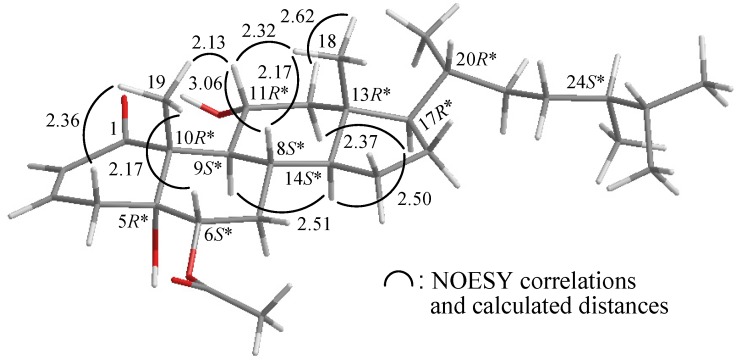
The computer-generated model of **2** using MM2 force field calculations and the calculated distances (**Å**) between selected protons with key NOESY correlations.

The configuration of two chiral centers (C-20 and C-24) in the side chain of **2** was elucidated by comparison of ^13^C NMR spectral data (*δ*_C_ 36.0, 39.0) with those of **3 **(*δ*_C_ 36.3, 39.1) [5], **4 **(*δ*_C_ 36.3, 39.1) [6]and (24*S*)-24-methylcholest-5-en-3β-ol (*δ*_C_ 36.26, 39.17) [7]. The configuration of the ring junctures in **2** was confirmed by NOESY analysis, as shown in Figure 5. The NOESY correlations between H-4β/Me-19; H-6/H_3_-19; H-8/H-11; H-8/Me-18; H-9/H-14; H-11/Me-18; H-11/Me-19; H-12β/Me-18; H-12α/H-17; and H-14/H-17 suggested that the 6-acetoxy and 11-hydroxy groups were α-oriented and all ring fusions in **2** were of a *trans* configuration. The coupling constants of H-6 and H-7a/b (*J* = 12.0, 5.5 Hz) suggested that H-6 was an axial hydrogen. This result further supported that the 6-acetoxy was α-oriented in **2**. Due to the fact that coupling pattern of H-11 in **2** appeared as a broad singlet in the ^1^H NMR spectrum of **2**, it is difficult to elucidate the relative stereochemistry of the 11-hydroxy group in **2** by vicinal coupling constant analysis; however, H-11 showed significant correlations with H-8, Me-18 and Me-19 in the NOESY analysis of **2**, which suggested that the 11-hydroxy group in **2** was α-oriented. 

**Table 2:** ^1^H (500 MHz, CDCl_3_) and ^13^C (125 MHz, CDCl_3_) NMR data, ^1^H–^1^H COSY and HMBC correlations for sterol **2**.

Position	*δ*_Η_ (*J* in Hz)	*δ*_C_,Mult.	^1^H–^1^H COSY	HMBC (H→C)
1		205.8, C		
2	6.15 dd (10.5, 2.5)	128.8, CH	H-3	n.o.
3	6.68 ddd (10.5, 5.5, 2.5)	140.7, CH	H-2, H_2_-4	n.o.
4a	2.48 dd (20.5, 5.5)	31.1, CH_2_	H-3, H-4β	C-2, -3, -5, -10
4b	2.91 br d (20.5)		H-3, H-4α	n.o.
5		78.4, C		
6	5.06 dd (12.0, 5.5)	75.1, CH	H_2_-7	C-5, -8, acetate carbonyl
7a	1.29 m	33.8, CH_2_	H-6, H-7b, H-8	C-6, -8, -9
7b	2.03 m		H-6, H-7a, H-8	C-9
8	1.27 m	29.2, CH	H_2_-7, H-9, H-14	n.o.
9	1.62 m	54.3, CH	H-8, H-11	C-10
10		54.3, C		
11	3.91 br s	66.9, CH	H-9, H_2_-12, OH-11	n.o.
12a	1.13, m	48.9, CH_2_	H-11, H-12β	C-11, -13, -14, -17, -18
12b	2.24 dd (12.5, 5.0)		H-11, H-12α	C-11, -13, -14, -17, -18
13		43.1, C		
14	1.19 m	54.9, CH	H-8, H_2_-15	C-13, -18
15	1.57 m	23.9, CH_2_	H-14, H_2_-16	n.o.
16	1.31 m; 1.89 m	28.1, CH_2_	H_2_-15, H-17	n.o.
17	1.15 m	55.9, CH	H_2_-16, H-20	C-13, -18
18	0.67 s	13.0, CH_3_		C-12, -13, -14, -17
19	1.34 s	9.8, CH_3_		C-1, -5, -9, -10
20	1.32 m	36.0, CH	H-17, H_3_-21, H_2_-22	C-22
21	0.89 d (6.5)	18.7, CH_3_	H-20	C-17, -20, -22
22a	0.91 m	33.5, CH_2_	H-20, H-22b, H_2_-23	C-20, -23, -24
22b	1.37 m		H-20, H-22a, H_2_-23	n.o.
23a	0.93 m	30.6, CH_2_	H_2_-22, H-23b, H-24	C-20, -22, -24
23b	1.36 m		H_2_-22, H-23a, H-24	C-22
24	1.20 m	39.0, CH	H_2_-23, H-25, H_3_-28	C-22
25	1.56 m	31.4, CH	H-24, H_3_-26, H_3_-27	C-24, -26, -27, -28
26	0.85 d (7.0)	20.5, CH_3_	H-25	C-24, -25, -27
27	0.78 d (6.5)	17.6, CH_3_	H-25	C-24, -25, -26
28	0.77 d (6.5)	15.4, CH_3_	H-24	C-23, -24, -25
OH-11	1.74 d (4.0)		H-11	n.o.
6-OAc		171.5, C		
	2.11 s	21.2, CH_3_		Acetate carbonyl

n.o. = not observed.

The *in vitro* anti-inflammatory effects of compounds **1** and **2** were tested (Table 3). 6-*epi*-yonarasterol B (**2**) was found to show significant inhibitory effects on the generation of superoxide anions and the release of elastase by human neutrophils. 

**Table 3:** Inhibitory effects of compounds **1** and **2** on the generation of superoxide anions and the release of elastase by human neutrophils in response to FMLP/CB.

Compounds	Superoxide Anions			Elastase Release	
	IC_50_ (µg/mL)	Inh % *^a^*		IC_50_ (μg/mL)	Inh % *^a^*
**1**	>10.0	2.52 ± 3.02		>10.0	1.83 ± 3.46
**2**	2.98 ± 0.29	89.76 ± 5.63		1.13 ± 0.55	95.54 ± 6.17
DPI *^b^*	0.82 ± 0.31				
Elastatinal *^b^*				31.82 ± 5.92	

*^a^* Percentage of inhibition (Inh %) at a concentration of 10 µg/mL; *^b^* DPI (diphenylene indoniumn) and elastatinal were used as reference compounds.

## 3. Experimental Section

### 3.1. General Experimental Procedures

Optical rotations were measured on a Jasco P-1010 digital polarimeter. Infrared spectra were recorded on a Varian Diglab FTS 1000 FT-IR spectrophotmeter; peaks are reported in cm^−1^. The NMR spectra were recorded on a Varian Mercury Plus 400 or on a Varian Inova 500 NMR spectrometer. Coupling constants (*J*) are given in Hz. ^1^H and ^13^C NMR assignments were supported by ^1^H–^1^H COSY, HMQC, HMBC and NOESY experiments. ESIMS and HRESIMS were recorded on a Bruker APEX II mass spectrometer. Column chromatography was performed on silica gel (230–400 mesh, Merck, Darmstadt, Germany). TLC was carried out on precoated Kieselgel 60 F_254_ (0.25 mm, Merck) and spots were visualized by spraying with 10% H_2_SO_4_ solution followed by heating. Normal phase HPLC was performed using a system comprised of a Hitachi L-7100 pump, a Hitahci L-7455 photodiode array detector, a Rheodyne injection port and a normal phase column (Hibar 250 × 10 mm, Merck, silica gel 60, 5 μm). Reverse phase HPLC was performed using a system comprised of a Hitachi L-7100 pump, a Hitahci L-2455 photodiode array detector, a Rheodyne injection port and a reverse phase column (Polaris 5 C18-A 250 × 10 mm, Varian, silica gel 60, 5 μm). 

### 3.2. Animal Material

Specimens of the gorgonian coral *Echinomuricea* sp. were collected by hand using scuba equipment off the coast of southern Taiwan and stored in a freezer until extraction. This organism was identified by comparison with previous descriptions [8,9]. A voucher specimen was deposited in the National Museum of Marine Biology and Aquarium, Taiwan.

### 3.3. Extraction and Isolation

The freeze-dried and minced material of *Echinomuricea* sp. (wet weight 1.68 kg, dry weight 428 g) was extracted with a mixture of methanol (MeOH) and dichloromethane (1:1). The residue was partitioned with ethyl acetate (EtOAc) and H_2_O. The EtOAc layer was partitioned between MeOH and *n*-hexane. The *n*-hexane layer was separated by silica gel and eluted using *n*-hexane/EtOAc/MeOH to yield 21 fractions A–U. Fraction L was separated on silica gel and eluted using *n*-hexane/EtOAc (stepwise, 50:1–pure EtOAc) to yield 16 fractions, L1–L16. Fraction L8 was purified by normal-phase HPLC using a mixture of *n*-hexane and EtOAc (8:1) as the mobile phase to afford compound **1** (0.9 mg). Fraction R was chromatographed on silica gel and eluted using *n*-hexane/EtOAc (stepwise, 1:1–pure EtOAc) to yield fractions R1–R13. Fraction R7 was separated by normal-phase HPLC using a mixture of *n*-hexane and acetone (4:1) as the mobile phase to afford 14 fractions R7A–R7N. Fraction R7M was further purified by reverse-phase HPLC using a mixture of methanol and H_2_O (85:15) to yield **2** (0.7 mg).

Echinolabdane A (**1**): yellowish oil; [α]23D +8 (*c* 0.03, CHCl_3_); IR (neat) ν_max_ 1765 cm^−1^; ^1^H (CDCl_3_, 400 MHz) and ^13^C (CDCl_3_, 100 MHz) NMR data, see Table 1; ESIMS: *m/z* 341 [M + Na]^+^; HRESIMS: *m/z* 341.2095 (calcd. for C_20_H_30_O_3_Na, 341.2093).

6-*epi*-Yonarasterol B (**2**): white powder; mp 93–94 C; [α]25D −22 (*c* 0.05, CHCl_3_); IR (neat) ν_max_ 3392, 1732, 1671 cm^−1^; ^1^H (CDCl_3_, 500 MHz) and ^13^C (CDCl_3_, 125 MHz) NMR data, see Table 2; ESIMS: *m/z* 511 [M + Na]^+^; HRESIMS: *m/z* 511.3396 (calcd. for C_30_H_48_O_5_Na, 511.3399).

### 3.4. Molecular Mechanics Calculations

Implementation of the MM2 force field [4] in CHEM3D PRO software from Cambridge Soft Corporation (Cambridge, MA, USA; ver. 9.0, 2005) was used to calculate the molecular models.

### 3.5. Cytotoxicity Testing

The cytotoxicity was assayed using a modification of the MTT [3-(4,5-dimethylthiazol-2-yl)-2,5-diphenyltetrazolium bromide] colorimetric method. Cytotoxicity assays were carried out according to previously described procedures [10,11].

### 3.6. Superoxide Anion Generation and Elastase Release by Human Neutrophils

Human neutrophils were obtained by means of dextran sedimentation and Ficoll centrifugation. Measurements of superoxide anion generation and elastase release were carried out according to previously described procedures [12,13]. Briefly, superoxide anion production was assayed by monitoring the superoxide dismutase-inhibitable reduction of ferricytochrome *c*. Elastase release experiments were performed using MeO-Suc-Ala-Ala-Pro-Valp-nitroanilide as the elastase substrate.

## 4. Conclusions

Labdane derivatives exist extensively in terrestrial plants [14], and compounds of this type have also been obtained from various marine organisms, including red algae belonging to the genus *Laurencia* [15,16,17,18,19]; sponges *Chelonaplysilla erecta* [20], *Raspaciona aculeata* [21], *Agelas* sp. [22]; and nudibranch *Austrodoris kerguelensis* [23]. It is worth noting that echinolabdane A (**1**) is the first labdane derivative to be isolated from marine organisms belonging to the phylum Cnidaria. The labdane analogue possessing a tetracyclic skeleton with an oxepane ring jointed to a γ-lactone ring by a hemiketal moiety, **1** (echinolabdane A), was discovered for the first time in this study. Furthermore, 6-*epi*-yonarasterol B (**2**) is the first steroid derivative to be isolated from a gorgonian coral belonging to the genus *Echinomuricea*, and this compound was found to exhibit significant anti-inflammatory activities. The gorgonian coral *Echinomuricea* sp. has begun to be transplanted in tanks for the extraction of natural products in order to establish a stable supply of bioactive material.
